# P-1706. Variability in Stool Multiplex PCR Test Utilization in the Urgent Care Setting

**DOI:** 10.1093/ofid/ofaf695.1878

**Published:** 2026-01-11

**Authors:** Timothy C Jenkins, Melody Zwakenberg, Margaret M Cooper, Katherine C Shihadeh, Michael Breyer, Cory Hussain, Laura Triplett, Lindsey Fish

**Affiliations:** Denver Health, Denver, Colorado; Denver Health, Denver, Colorado; Denver Health, Denver, Colorado; Denver Health, Denver, Colorado; Denver Health, Denver, Colorado; Sutter Bay West Medical Group, San Francisco, California; Denver Health Medical Center, Denver, Colorado; Denver Health, Denver, Colorado

## Abstract

**Background:**

Overuse of a stool multiplex PCR test in low-yield clinical scenarios may lead to identification of non-pathogenic or colonizing organisms, unnecessary antibiotic use, and significant expense. Understanding provider utilization patterns and outlier users of this test may assist in developing interventions to promote its appropriate use. The objectives of this study were to evaluate the variability in stool PCR test ordering among urgent care providers and evaluate factors associated with high utilization.

**Figure:**
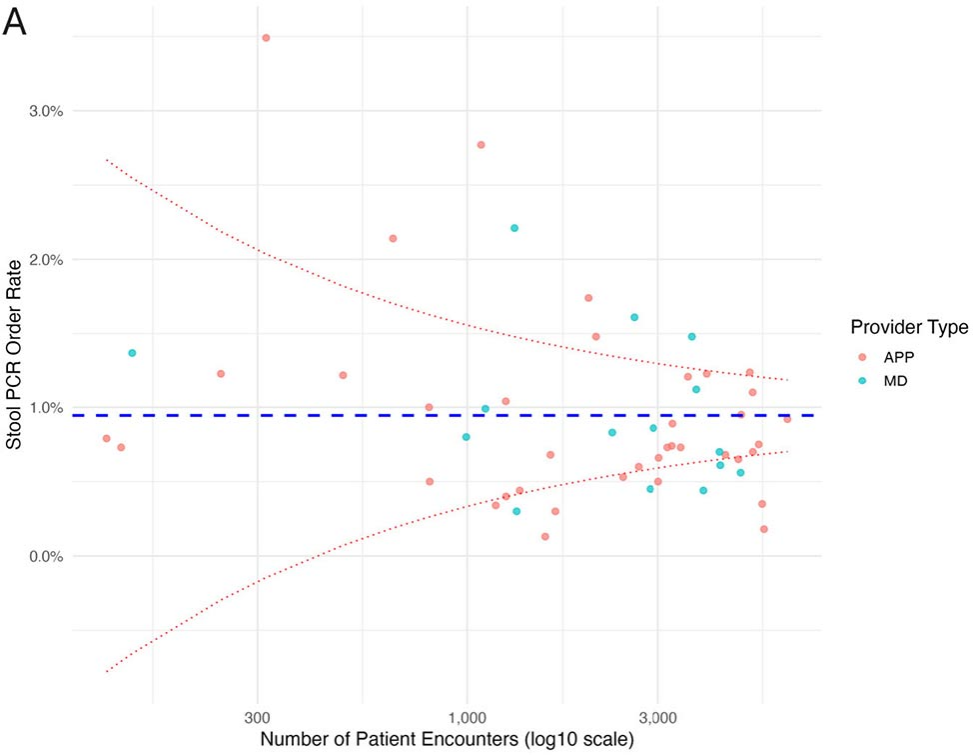
Funnel plot of stool PCR ordering by individual providers overall Each dot represents an individual provider. The X-axis is the number of patient encounters during the study period and the Y-axis is the percent of encounters where a stool PCR was ordered. The dashed blue line is the mean order rate. The dashed red lines are two standard deviations from the mean. APP, advanced practice provider; MD, physician.

**Figure:**
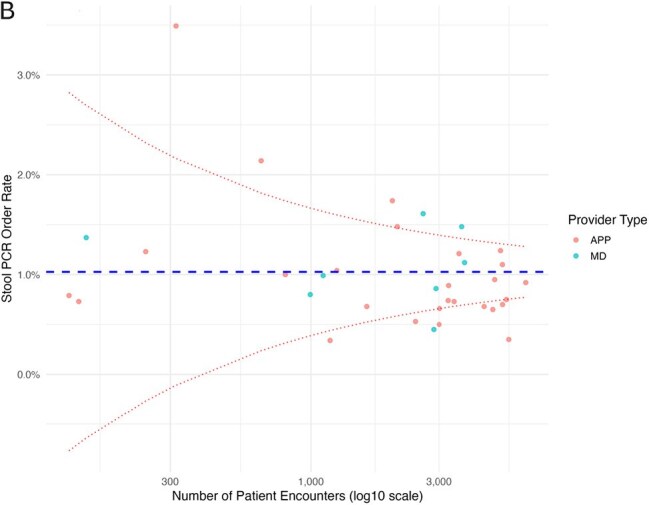
Funnel plot of stool PCR ordering by individual providers in Urgent Care A Each dot represents an individual provider. The X-axis is the number of patient encounters during the study period and the Y-axis is the percent of encounters where a stool PCR was ordered. The dashed blue line is the mean order rate. The dashed red lines are two standard deviations from the mean. APP, advanced practice provider; MD, physician.

**Methods:**

This was a retrospective study of patients with an encounter to two urgent care centers in an academic, integrated healthcare system from 7/1/2022 – 6/30/2024. The primary outcome was the stool PCR order rate, defined as the number of stool PCR orders divided by the number of patient encounters, expressed as a percentage. The order rate was calculated overall, by urgent care site, by provider years in clinical practice (< 5 years vs ≥ 5 years), by provider type (APP vs physician), and for individual providers. For individual providers, an order rate of two or more standard deviations above the mean was defined as outlier use.

**Figure:**
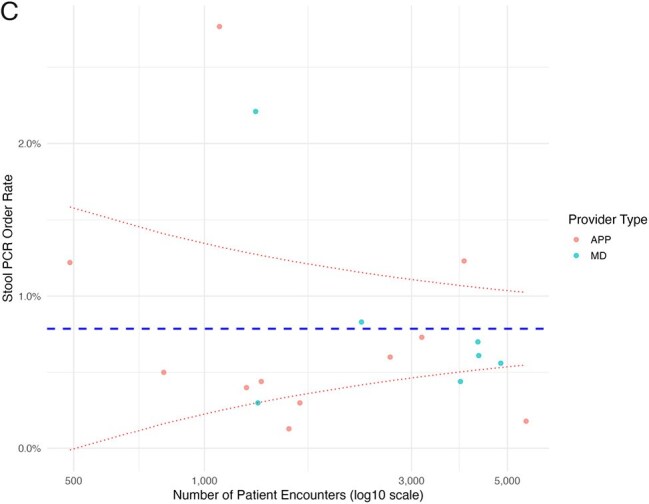
Funnel plot of stool PCR ordering by individual providers in Urgent Care B Each dot represents an individual provider. The X-axis is the number of patient encounters during the study period and the Y-axis is the percent of encounters where a stool PCR was ordered. The dashed blue line is the mean order rate. The dashed red lines are two standard deviations from the mean. APP, advanced practice provider; MD, physician.

**Results:**

Of 142,805 patient encounters completed by 53 providers, the stool PCR was ordered in 1,186 (overall order rate 0.8%). The order rate was significantly higher at Urgent Care A vs B (0.9 vs 0.7%, respectively, p< .001). APPs and physicians ordered the test at similar rates (0.8% vs 0.9%, respectively, p=0.36). Providers with < 5 years of experience had a significantly higher order rate than those with ≥ 5 years of experience (1.0% vs 0.8%, respectively, p< .001). Among individual providers, substantial variability in order rates was observed overall (Figure A). At Urgent Care A, there was a 10-fold difference between the highest and lowest order rate (3.5% vs 0.3%, respectively) (Figure B). At Urgent Care B, there was a 14-fold difference between the highest and lowest order rate (2.8% vs 0.2%) (Figure C). In total, 10 (19%) providers were outlier users, accounting for 25% of all orders.

**Conclusion:**

In the urgent care setting, there is substantial variability in stool multiplex PCR test utilization at the provider level. Developing standardized criteria for use and/or targeting outlier users may be effective approaches to reduce low-yield or unnecessary tests.

**Disclosures:**

All Authors: No reported disclosures

